# Spatial Characteristics of Tree Diameter Distributions in a Temperate Old-Growth Forest

**DOI:** 10.1371/journal.pone.0058983

**Published:** 2013-03-19

**Authors:** Chunyu Zhang, Yanbo Wei, Xiuhai Zhao, Klaus von Gadow

**Affiliations:** 1 Key Laboratory for Forest Resources and Ecosystem Processes of Beijing, Beijing Forestry University, Beijing, China; 2 Faculty of Forestry and Forest Ecology, Georg-August-University Göttingen, Göttingen, Germany; The Ohio State University, United States of America

## Abstract

This contribution identifies spatial characteristics of tree diameter in a temperate forest in north-eastern China, based on a fully censused observational study area covering 500×600 m. Mark correlation analysis with three null hypothesis models was used to determine departure from expectations at different neighborhood distances. Tree positions are clumped at all investigated scales in all 37 studied species, while the diameters of most species are spatially negatively correlated, especially at short distances. Interestingly, all three cases showing short-distance attraction of *dbh* marks are associated with light-demanding shrub species. The short-distance attraction of *dbh* marks indicates spatially aggregated cohorts of stems of similar size. The percentage of species showing significant *dbh* suppression peaked at a 4 m distance under the heterogeneous Poisson model. At scales exceeding the peak distance, the percentage of species showing significant *dbh* suppression decreases sharply with increasing distances. The evidence from this large observational study shows that some of the variation of the spatial characteristics of tree diameters is related variations of topography and soil chemistry. However, an obvious interpretation of this result is still lacking. Thus, removing competitors surrounding the target trees is an effective way to avoid neighboring competition effects reducing the growth of valuable target trees in forest management practice.

## Introduction

A forest is composed of a set of trees, which are characterized by their locations and sizes. Tree diameter distributions can provide the necessary information about tree sizes, ignoring tree locations. Tree diameters are, however, associated with tree positions and growth is sensitive to spatial interaction between trees [1]–[2] as well as local habitat characteristics [3]. Continuous functions of the spatial coordinates including tree size have been implemented in a method known as Mark Correlation analysis, in which tree locations and diameters are regarded as a realization of a marked point stochastic process [4]–[7].

Spatial dependence of topography, drainage and soil characteristics can create different spatial structures of a forest community [8]. Most processes affecting forest trees occur at short neighborhood distances, such as seed dispersal, some pollination and competition for light and nutrients [9]–[11]. Forest soils provide nutrition and moisture for tree growth and the spatial heterogeneity of soil nutrients will thus affect the spatial structure of tree diameter distributions at particular spatial scales. Specific null models, such as a random labeling model and a heterogeneous Poisson model, are required to factor out the effects of habitat associations at varying spatial scales.

The theory of marked point processes provides a formal framework for analysis of spatial characteristics of tree diameter distributions, in which the points indicate tree locations, and the marks might denote particular tree characteristics, such as diameter at breast height (*dbh*), tree height and growth during a given time span [6], [12]–[13]. Assessment of the proportion of species exhibiting aggregation, regularity or randomness of *dbh* at different spatial scales provides important insights into the spatial structures of temperate communities. Studies investigating neighborhood effects indicated that direct plant–plant interactions worked strongly at local plant neighborhoods but faded away at larger scales in tropical forests [14]–[16] and temperate forests [7], [17]. We therefore assume departures from expectations at local neighborhood distances while assuming randomness at larger distances.

These concepts were applied to data from a fully censused 30ha old-growth forest plot, in which the tree locations were mapped and the soil chemistry and topography were available spatially explicit. The objectives of this study are (*i*) to ascertain the spatial characteristics of species-specific *dbh*'s at various distances, and (*ii*) to assess the particular effects of habitat heterogeneity.

## Materials and Methods

### Ethics statement

All necessary permits were obtained for the field studies. The study was approved by the Ethics Committee of *Jiaohe* Administration Bureau of the Forest Experimental Zone in Jilin province, in Northeastern China.

### Field sample

The observations for this study were obtained in a fully censused, unmanaged old-growth forest plot (OGF), covering 30 ha (500×600 m). The OGF plot was established in a temperate mixed broadleaf-conifer forest in the summer of 2010, and is located at 43°57.928′ ∼ 43°58.214′N, 127°45.287′ ∼127°45.790′E. The OGF study area is situated in a protected locality at *Jiaohe* Administration Bureau of the Forest Experimental Zone, far away from villages, where human disturbance has been virtually unknown. The elevation of the OGF plot ranges from 576 to 784 m above sea level and the topography is characterized by a valley between two slopes. Altogether 37 tree species with more than 20 individuals are included in this analysis (Table S1).

### Point pattern analysis

Habitat heterogeneity and plant interactions may cause increasing variation of the local point densities. Accordingly, *Ripley*'s *L*(*r*) functions were used to analyze the spatial distribution of point positions [18]–[19]. To correct edge effects, each count was weighted by the inverse of the proportion of the circle that falls within the study plot [18], [20]. *L*(*r*) is defined as follows:
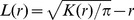
(1)where, *K*(*t*) is *Ripley*'s univariate *K-*function [21], and *r* is the distance category.

### Marked point pattern and null hypothesis models

The spatial correlations of tree diameters were determined using the mark correlation function (MCF). The mark correlation function takes account of a quantitative characteristic (such as tree breast height diameter in this study) which is associated with tree locations, and then calculates the spatial correlation of these marks in the observed point pattern. The spatial correlation of marks in the marked point process is characterized by a test function *k[mm](r)* for two marks *M_i_* and *M_j_*. This test function describes the correlation between the marks *M_i_* and *M_j_* at different points which are a distance *r* apart. According to Stoyan and Stoyan [22], for a point process *X* with numeric marks (*dbh*), the mark correlation function *k[mm](r)* is defined as:
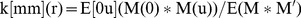
(2)where, *E[0u]* denotes the conditional expectation given that there are points of the process at the locations *0* and *u* separated by a distance *r*, and where *M(0),M(u)* denote the marks attached to these two points. *M*, *M*' are random marks drawn independently from the marginal distribution of marks, and *E* is the expectation.

The implemented edge correction method is *Ripley*'*s* isotropic correction [22]. Three null hypothesis models were used to compute the confidence intervals. When the observed *k[mm](r)* was greater than, equal to, or less than the 99% confidence interval calculated from the predicted *k[mm](r)* from 99 realizations of the adopted model, the *dbh* values of the focal species were assumed to be positive, independent or negative correlation, respectively.

One of the adopted models is a homogeneous Poisson model (denoted by *HomP*). In the homogeneous Poisson process, the assumption is that the spatial location of a given point (tree) is independent of any other points (trees). Thus, *HomP* was used to examine the effect of a pure random process. This process implies identical average tree density per unit area for one given focal species. The locations of the focal species were randomized using 99 realizations of the homogeneous Poisson process. Then, the observed values of the marks (*dbhs*) were randomly assigned to the completely random locations.

The second model that was used in this study is a random labeling model (*RLM*) which assumes that environmental conditions play a decisive role in the spatial distribution of the trees. The observed values of the marks (*dbhs*) were assigned randomly to the positions of the focal species, while the positions were kept unchanged. Then, 99 random labeling realizations were generated to obtain confidence envelopes.

In addition, a heterogeneous Poisson model (*HetP*) was used to examine the effect of habitat heterogeneity. The *HetP* process assumes that the density of each tree species is associated with the specific habitat conditions in the study area. Thus, the *dbh* values are randomized among trees, while the tree positions are simulated and distributed in accordance with the intensity of the focal species. The pixel image of the tree density of the focal species was estimated using a Gaussian kernel. 99 realizations of the inhomogeneous Poisson process with intensity equal to the pixel values of the image were generated to obtain confidence envelopes.

Significant departure from the above three null models was determined using the lowest and highest value of the 99 simulations of the null models to generate 99% simulation envelopes.

### Soil chemical properties

Soil sampling included lattice sampling and random sampling to assess the spatial variation of soil chemical properties at different spatial scales (for details see Figure S1). A total of 540 sampling position was available in the 30ha plot. The soil samples were taken from the upper (0–10 cm), middle (10–20 cm) and lower layer (>20 cm) to analyze total nitrogen (using the Kelvin Digestion method), total potassium (fusion with NaOH method), organic matter (exterior heating potassium dichromate-density method) and soil pH in August of 2009.

A semivariogram, which indicates spatial correlations in observations measured at sample locations, was used to fit a model of the spatial correlations of the soil chemical properties. Box-Cox transformation was applied for above soil chemical variables to meet the normality assumptions required in the semivariogram model [23] (Table S2). Several theoretical models were used to simulate the spatial variance of soil chemical properties (Table S3). On the basis of the optimizing models, kriging interpolation was used to estimate the total nitrogen, total potassium, organic matter and pH value for each tree position. Pearson's correlation analysis was used to measure the relationships between tree diameter and soil chemical properties.

### Diameter differentiation and topography

The 30-ha study area was subdivided into 750 cells, each covering 20×20 m (400 m^2^). Then the coefficient of variation of tree diameters (*CVd* = *dbh* standard deviation/mean *dbh*) was calculated for all trees with a *dbh* greater or equal to 1 cm in each cell. The spatial distribution of *CVd*'*s* is shown in Figure S2.

To quantify topography, the heights at the intersections of the cell grid lines, called “nodes”, were measured. The elevation of a particular cell was calculated as the mean of the elevations of its four corner nodes. The cell slope for each of the five cell sizes was estimated as the mean angular deviation from the horizontal plane of each of the four triangular planes which were formed by connecting three of its adjacent corners [24]. The convexity of a cell was calculated as the elevation of the focal cell minus the mean elevation of the eight surrounding cells [25]. For the edge cells, convexity was taken as the elevation of the center point minus the mean of the four corners. Positive and negative convexity values respectively indicate convex (ridge) and concave (valley) land surfaces. The aspect of a cell can be obtained from the average angle of the four triangular planes that deviate from the north direction. Pearson's correlation coefficient was used to test the correlations between *CVd* and four topographical factors.

All calculations were performed using the “spatstat” and “geoR” packages in the comprehensive *R* environment [26]. The main code sections used in our analyses are included in the attached Text S1.

## Results

### Spatial characteristics of diameter distributions

**Figure 1:**
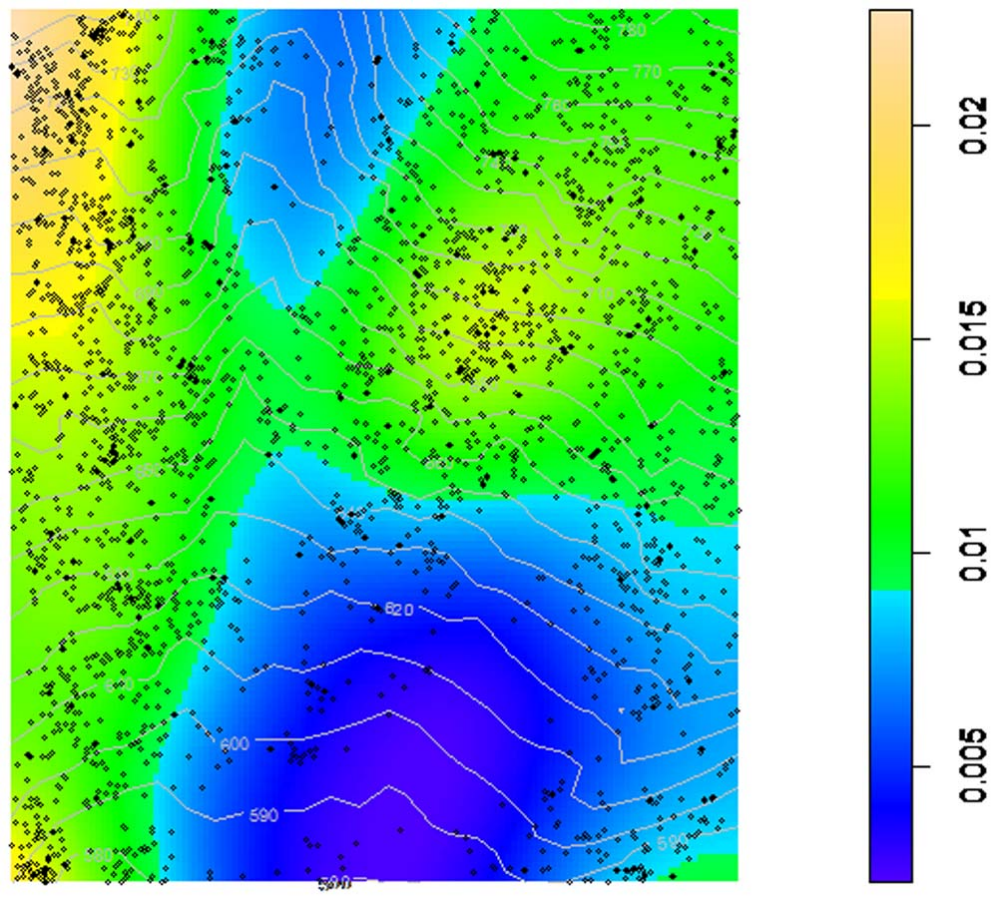
Example of a kernel smoothed intensity map showing the point pattern. The map colours show the intensities (number of trees per m^2^) of *Syringa reticulata* var. *amurensis* and the elevation contours at 10-m intervals within the 30-ha study area. The unit of the axes is meters.

To analyze the spatial characteristics of the diameter distributions of each of the 37 tree species, the heterogeneous Poisson process (*HetP*) was used, involving three steps. First, for each of the tree species, the pixel image of the tree density was estimated using a Gaussian kernel. Figure 1 is an example of the kernel smoothed intensity map of the point distribution for the species *Syringa reticulata* var. *amurensis*. The tree locations were then simulated and distributed in accordance with the intensity of the focal species. Finally, the *dbh* values were randomly assigned to the simulated tree locations.

**Figure 2:**
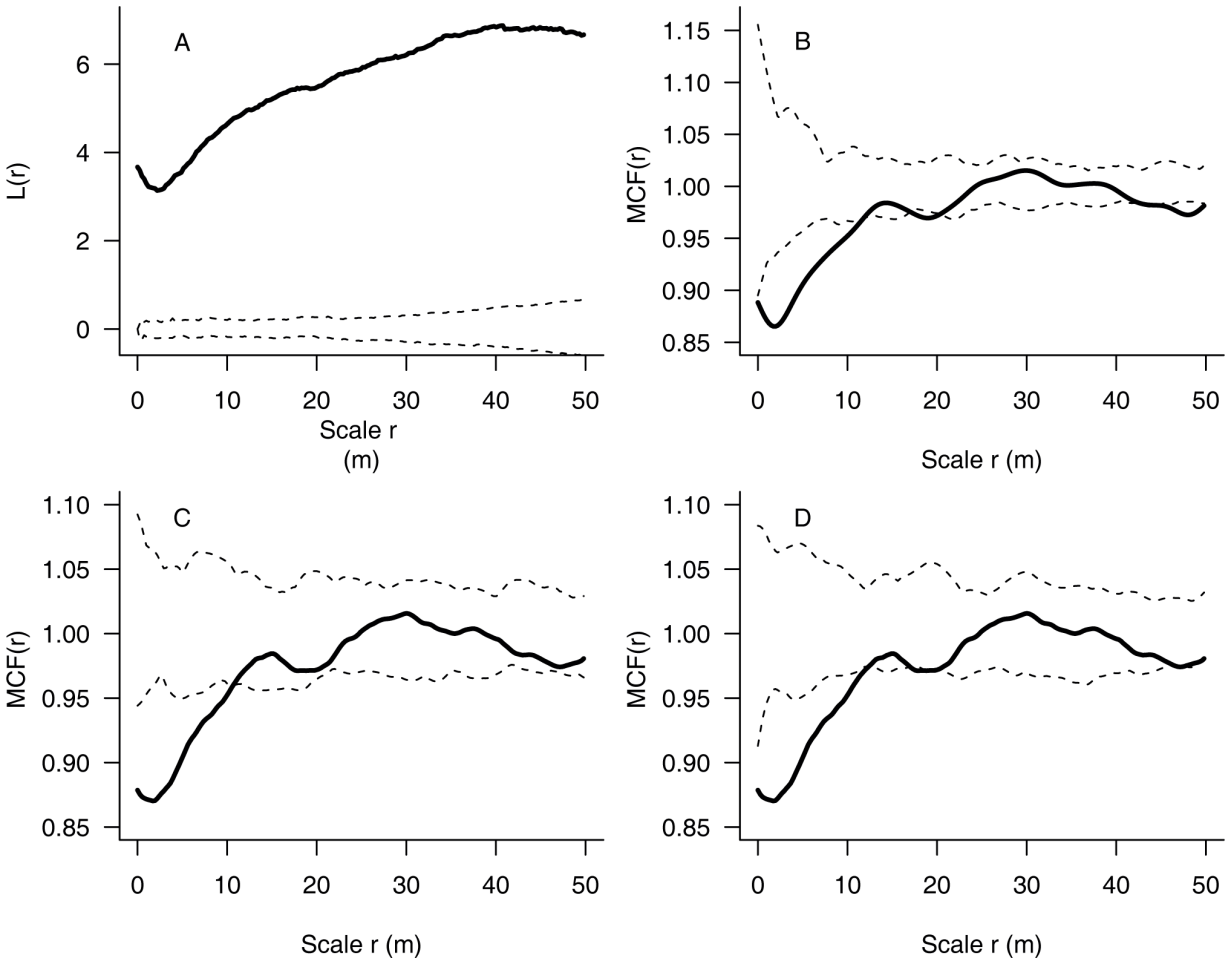
Exemplary results for *Syringa reticulata* var. *amurensis* to illustrate the analysis. Diagram a presents the spatial distribution pattern using the *L* function. Diagrams b, c and d show the spatial characteristics of the diameter distribution evaluated by the mark correlation function. The significance of a and b was tested by the homogeneous Poisson model, c by the random labeling model, and d by the heterogeneous Poisson model. Dashed lines indicate the confidence envelopes, while solid lines indicate the *L* or *MCF* values calculated from the observations. When the solid line was below the lower envelope, inside both envelopes, or above the upper envelope, the pattern was assumed to be significantly regular, spatially random, or significantly aggregated in the *L*-function analysis. Correspondingly, a solid line above the upper envelope, inside both envelopes, or below the lower envelope, indicates significant positive, independent, or negative correlation of *dbh* marks in the *MCF* analysis.

The spatial characteristics of the tree locations were then evaluated using the complete spatial randomness model while their *dbh* marks were analysed using the *HomP*, *RLM* and *HetP* models. The results for the species *Syringa reticulata* var. *amurensis* show, for example, that tree positions were significantly aggregated at distances between 0 and 50 m. The *dbh*'s, however, are spatially negatively correlated at much smaller distances: between 0 and 7 m using the *HomP* model; between 0 and 11 m using the *RLM* model and between 0 and 10 m using the *HetP* model (Figure 2). Interestingly, the results of the three models are very similar for the example species. However, this is not always the case. For example, the results differ greatly in *Betula platyphylla.*

### Scale-dependent distribution of species and diameters

The spatial point patterns of each species were analysed using *Ripley*'*s L*-function to test departure from complete spatial randomness. The positions of all 37 species showed significant aggregation, even at greater distances up to 50 m (Table 1).

**Table 1:** Spatial characteristics of tree locations and tree *dbhs* at 0–50 m distances.

Species name	Lightshade	Canopyundergrowth	*L* function at 0–50 m	Marked point pattern at different distances		
				Homogeneous Poisson model (*HomP*)	Random labeling model (*RLM*)	Heterogeneous Poisson model (*HetP*)
*Betula platyphylla*	L	C	0–50 (+)	0–50 (r)	0–50 (r)	0–6 (−)
*Acer mandshuricum*	L	C	0–50 (+)	0–50 (−)	0–32 (−)	0–42,47–50 (−)
*Syringa reticulata var. amurensis*	L	U	0–50 (+)	0–7 (−)	0–11 (−)	0–10 (−)
*Euonymus macropterus*	L	U	0–50 (+)	20–28 (+)	0–16,20–29 (+)	0–15,20–30,39–46 (+)
*Padus racemosa*	L	U	0–50 (+)	8–26,41–50 (−)	0–26 (−)	0–27 (−)
*Abies nephrolepis*	S	C	0–50 (+)	0–50 (r)	0–50 (r)	0–50 (r)
*Ulmus davidiana var. japonica*	L	C	0–50 (+)	0–50 (r)	0–3 (−)	0–8 (−)
*Acanthopanax senticosus*	S	U	0–50 (+)	0–50 (r)	0–5 (+),35–50 (−)	0–15 (+),35–50 (−)
*Acer barbinerve*	S	U	0–50 (+)	22–29,32–34,42–50 (−)	44–50 (–)	43–50 (−)
*Ulmus macrocarpa*	L	C	0–50 (+)	0–50 (r)	0–15 (−)	0–15 (−)
*Philadelphus schrenkii*	M	U	0–50 (+)	0–50 (r)	0–50 (r)	0–50 (r)
*Betula costata*	L	C	0–50 (+)	0–5 (−)	0–7 (−)	0–7 (−)
*Betula dahurica*	L	C	0–50 (+)	0–50 (r)	0–50 (r)	0–2 (−)
*Cerasus maximowiczii*	S	U	0–50 (+)	0–22 (+)	0–19 (+)	0–20 (+)
*Pinus koraiensis*	L	C	0–50 (+)	41–50 (−)	0–5,17–22,41–50 (−)	0–5,15–25,35–50 (−)
*Juglans mandshurica*	L	C	0–50 (+)	0–2 (−)	0–6 (−)	0–6 (−)
*Acer ukurunduense*	L	U	0–50 (+)	0–50 (r)	0–50 (r)	0–50 (r)
*Sorbus pohuashanensis*	L	U	0–50 (+)	0–50 (r)	0–10 (−)	0–12 (−)
*Fraxinus rhynchophylla*	L	C	0–50 (+)	0–50 (r)	36–42 (−)	0–50 (r)
*Phellodendron amurense*	L	C	0–50 (+)	48–50 (+)	0–50 (r)	0–50 (r)
*Lonicera praeflorens*	M	U	0–50 (+)	0–50 (r)	0–50 (r)	0–50 (r)
*Lonicera maackii*	L	U	0–50 (+)	39–50 (+)	0–14 (−)	0–17 (−)
*Tilia mandshurica*	L	C	0–50 (+)	0–50 (r)	18–45 (−)	18–45 (−)
*Ulmus laciniata*	L	C	0–50 (+)	0–50 (−)	0–50 (−)	0–50 (−)
*Euonymus pauciflorus*	L	U	0–50 (+)	5–14,19−26 (+)	0–26 (−)	0–26 (−)
*Aralia elata*	M	U	0–50 (+)	0–50 (r)	0–50 (r)	0–50 (r)
*Corylus mandshurica*	S	U	0–50 (+)	15–50 (−)	17–26,48–50 (−)	17–50 (−)
*Quercus mongolica*	L	C	0–50 (+)	0–50 (r)	0–50 (r)	0–50 (r)
*Carpinus cordata*	L	C	0–50 (+)	0–50 (−)	0–41 (−)	0–45 (−)
*Acer tegmentosum*	S	U	0–50 (+)	0–50 (r)	0–4 (−)	0–4 (−)
*Acer mono*	L	C	0–50 (+)	0–24 (−)	0–19 (−)	0–24 (−)
*Abies holophylla*	S	C	0–50 (+)	3–8 (−)	0–12 (−)	0–12 (−)
*Rhamnus davurica*	S	U	0–50 (+)	0–50 (r)	0–50 (r)	0–50 (r)
*Fraxinus mandshurica*	L	C	0–50 (+)	0–50 (r)	0–4 (−)	0–4 (−)
*Sorbus alnifolia*	M	C	0–50 (+)	0–50 (r)	0–50 (r)	0–50 (r)
*Populus koreana*	L	C	0–50 (+)	0–50 (r)	36–46 (−)	4–7,36–46 (−)
*Tilia amurensis*	L	C	0–50 (+)	0–50 (r)	3–11 (−)	3–11 (−)

Note: L means light; S means shade and M means middle. C means canopy trees and U means understory.

Spatial point patterns were tested for randomness using the *L*-function. The spatial characteristics of tree *dbhs* were analyzed by the homogeneous Poisson (*HomP*), random labeling (*RLM*) and heterogeneous Poisson (*HetP*). Spatial distances at which tree locations show significant aggregation, regularity and randomness are indicated by the symbols “+” in parenthesis in *L*-function. Spatial distances at which tree locations and tree *dbh* marks show significant positive, independent and negative correlation are indicated by the symbols “+”, “*r*” and “−” in parenthesis, respectively.

For each species, the spatial distribution of tree diameters was analysed separately (see Table 1). For most species, the results of the *HomP* model, which only simulates random points without changing *dbh*'s, are significantly different from those of the *RLM* and *HetP* models. In the *RLM* approach, the observed values of the marks are assigned randomly to tree positions (which are kept unchanged), assuming that the spatial distribution is influenced by habitat. The *HetP* model is also designed to take into account the effect of habitat heterogeneity by randomizing the *dbh* values, while the tree positions are simulated in accordance with the intensity of the focal species. These basic differences between *HomP* one the one hand and *RLM* and *HetP* on the other hand, explains the different results in Table 1.

**Figure 3:**
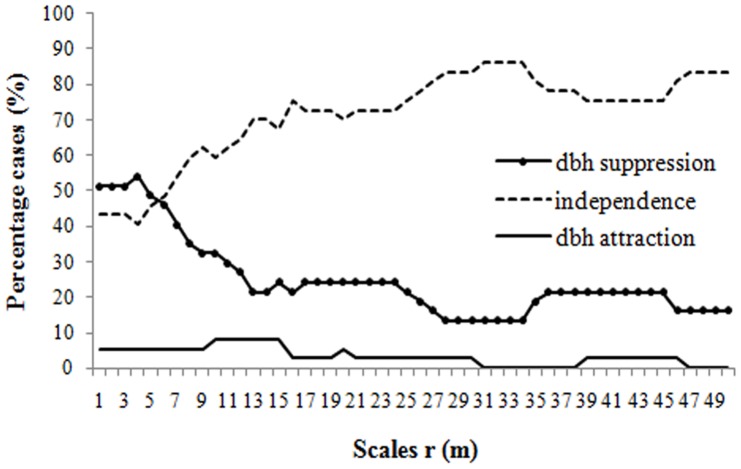
Proportion of species exhibiting significant departures from randomness. Diagram presents the proportion of species showing significant suppression of *dbh* marks (lines with solid circles), attraction of *dbh* marks (solid lines) and independence of *dbh* marks (dashed lines) under the heterogeneous Poisson model. For each distance, the three values add up to 100.

While tree positions are clumped at all studied scales, the diameters associated with them are significantly negatively correlated, especially at short distances, for most species under the *RLM* (24 of 37 species) and *HetP* model (25 of 37 species). At relatively small distances, the mark correlation function for three species, *Cerasus maximowiczii*, *Euonymus macropterus* and *Acanthopanax senticosus*, shows significant positive correlation. Positive correlations of *dbh* marks may reflect the effects of historical gap formation of these shrub species. Figure 3 further highlights the distance-dependent effects of the spatial distribution of tree *dbh*'s by presenting the proportions of positive, independent and negative effects at distances of up to 50 m for all species combined. The observational area is very large and it would be possible to evaluate distances up to 200 m. However, distances exceeding 50 m are not considered to be relevant.

Negative correlation indicates inhibition and suppression. For all tree species, the percentage of species showing significant *dbh* suppression peaks at a distance of 4 m, and decreases sharply with increasing distances between 4 to 13 m distances under the *HetP* model. In contrast, the percentage of species showing *dbh* independence increases with increasing distance (Figure 3).

### Effects of soil chemistry and topography

**Figure 4:**
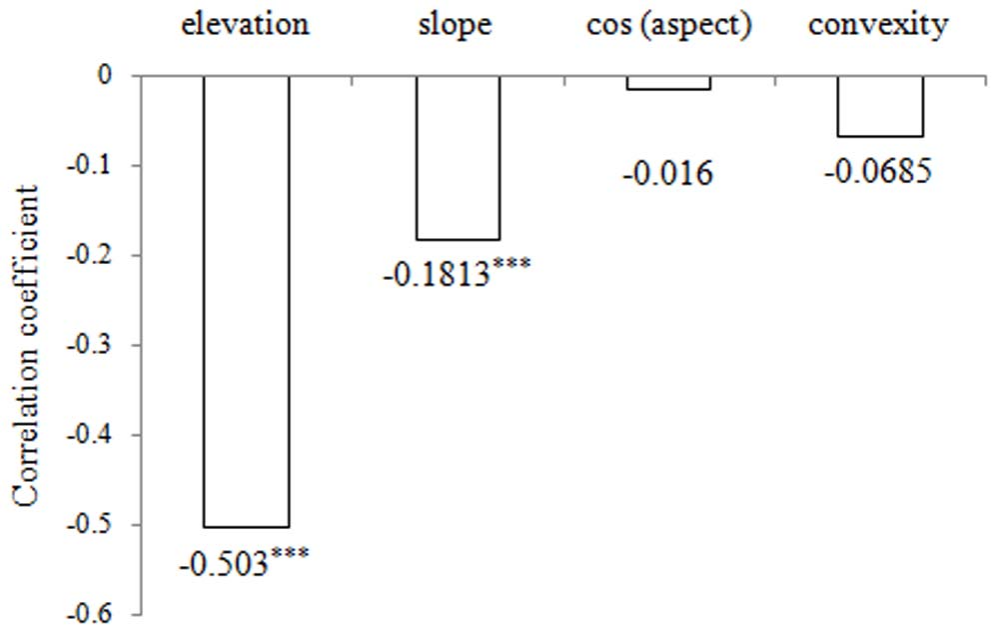
Pearson's correlations between the coefficient of variation of tree diameters and topographical variables. ^***^ indicated a significance at the 0.001 level.

Total nitrogen, total potassium, organic matter and the soil pH value of three soil layers showed significant spatial heterogeneity in the study area. Table S3 shows the semivariogram models of soil chemical properties in the study area while Figure 4 presents the semivariogram maps of the soil chemical properties which present the basis for kriging and the spatial estimates of total nitrogen, total potassium, organic matter and pH value for each tree position. Figure S3 shows the contour plots for soil chemical properties, based on the kriging models in Figure S4. There are some significant relationships between tree diameters and specific soil properties (Table S4). However, the underlying causes for these correlations are not known.

Pearson's correlation analysis was used to quantify the relationships between the coefficient of variation of tree diameters (*CVd*) and four topographical variables in the 20 m×20 m cells. Figure 4 shows that *CVd* is significantly associated with elevation and slope but not with aspect and convexity.

## Discussion

### Species aggregation and diameter suppression

Previous studies in subtropical and tropical forests have reported that individual tree species tend to be spatially aggregated and the degree of aggregation is scale dependent [27]–[31]. This has also been observed in temperate forests [32]–[33]. The spatial aggregation of tree species is usually thought to be caused by seed dispersal limitation [34] and environmental heterogeneity [32], [35]–[36]. In this study, all 37 species were found to be spatially clumped at all investigated scales from 0 to 50 metres. This result is not entirely unexpected if one assumes that the regeneration of seedlings is clustered in the vicinity of parent trees. Only long-term observation can reveal whether such clustering will be sustained over several tree generations. Habitat heterogeneity may also cause spatial aggregation, at least for particular species that show specific habitat preferences.

Neighborhood competition is an important characteristic of stand structure which can be defined as an interaction between neighboring individuals for territory or specific resources. The available resources are usually concentrated on an impact zone surrounding the trees. The bigger the tree, the greater the impact zone. Thus, the competition between two trees depends on their sizes and the distance between them. The “competition effect” between any two trees will increase with increasing size of the competitor and decrease with increasing tree-to-tree distance. Schlather et al. [37] tested the negative interactions in a spruce stand in the Fichtelgebirge, in northern Bavaria. He found a competitive radius of up to 6 m when using stem diameters as marks. A neighborhood competition effect up to a range of about 6 m was also found when using upper crown surface areas as marks in two deciduous and two coniferous forests in central Germany [38]. Our study also identified mostly independence of *dbh*'s at greater distances, but highly significant negative correlation at short distances.

All significant short-distance attraction of *dbh*'s was surprisingly associated with the light-demanding shrub species (for example, *Euonymus macropterus*, *Acanthopanax senticosus* and *Cerasus maximowiczii*). An obvious explanation for the spatially aggregated cohorts of stems of similar size would be that after establishment of a canopy gap, the open area is rapidly colonised by these light-demanding shrubs. These gap colonisation cohorts are likely to have similar diameters. However, the question remains why the tree size-clumping mainly occurs at short distances. Large-scale fires do not occur in the area and large scale wind damage is also unknown. Our study was carried out in an old-growth forest, in which most trees in the canopy layer are more than 150 years old. The old-growth forest has a mix of tree ages due to a distinct regeneration pattern. The uneven-aged structure often indicates that the forest represents a relatively stable ecosystem in the long term. Canopy gaps are essential in creating and maintaining mixed-age stands.

Research concerning the forest gap structure in this particular study area showed that almost all gaps have areas less than 1000 m^2^ [39]. A circular gap which occupies 1000 m^2^, has a radius of 17.8 m. Therefore, a likely explanation for the short distance clumping of *dbh*'s is that gaps are small, i.e. caused by the death and decay of large trees.

### Effects of soil chemistry and topography

Environmental variables such as ground cover vegetation, light conditions, microclimate, soil characteristics and ecological history play an important role in the spatial distribution of tree diameter at the study site. Topography and soil chemistry may significantly affect the spatial distribution of particular tree species. Valencia et al. [35], for example, found that tree aggregation patterns in tropical forests could be related to specific topographic features. Zhang et al. [40] could identify similar species-habitat associations in a 660×320 m temperate forest in north-eastern China. This study was based on the assumption that habitats are spatially autocorrelated and that species are spatially aggregated due to seed dispersal limitations. The relationships between species richness and topographic variables were found to be scale-dependent, while the great majority of the species showed distinct habitat-dependence in that study.

Regarding the effects of soil chemistry, John et al. [41] compared distribution maps of 10 essential plant nutrients in the soils to species maps of all trees to test plant-soil associations in three neotropical forest plots. They found that the spatial distributions of 36–51% of the tree species showed strong associations with soil nutrient distributions. Zhang et al. [42] determined the effects of different soil chemical variables on the variation of tree sizes in three study areas of uniform topography, each covering an area of 5.2ha and representing a specific forest developmental stage in the Changbai Mountain region. The results showed that over 14 percent of the spatial variation of tree diameters could be explained by soil chemistry in two secondary forests, and only 4.2 percent in the virgin forest.

These studies have shown effects of soil chemistry and topography on the tree distribution, but several questions regarding the effects on the spatial variation of tree diameters remain unanswered. The results of this study show that there are some significant relationships between tree diameters and specific soil properties (Table S4). For example, total N in the upper soil layer is positively correlated with the *dbh*'s of canopy species like *Acer mandshuricum*, *Ulmus macrocarpa*, *Philadelphus schrenkii*, *Juglans mandshurica*, *Acer ukurunduense*, *Acanthopanax senticosus*, *Fraxinus rhynchophylla* and *Carpinus cordata*. The correlations are highly significant and positive in the case of the last-named three species and negative in the others. There are also highly significant correlations between total K in the upper soil layer and the diameters of *Acer mandshuricum*, *Syringa reticulata* var. *amurensis*, *Acer barbinerve*, *Ulmus macrocarpa*, *Betula costata*, *Cerasus maximowiczii*, *Pinus koraiensis*, *Juglans mandshurica*, *Acer ukurunduense*, *Carpinus cordata*, *Acer tegmentosum*, *Acer mono*, *Sorbus alnifolia* and *Populus koreana*. Negative correlations between the pH value in the lower soil layer and tree diameters, which are also highly significant, are also found in *Syringa reticulata* var. *amurensis*, *Euonymus macropterus*, *Padus racemosa*, *Acer barbinerve*, *Pinus koraiensis*, *Carpinus cordata* and *Ulmus macrocarpa*.

The small-scale variations of soil chemical variables may have some influence on the spatial distribution of tree diameters. Suppression of *dbh*'s at close range may be caused by specific topographical and soil properties. However, these results are difficult to interpret. A young tree growing on a nutrient rich spot in a shaded and suppressed position will be small when compared with an old tree growing in a less favorable location. Tree diameters are related to tree age and past competition effects. Past interactions between neighboring trees would have influenced tree diameters. Furthermore, different species have significantly different growth rates. For example, light demanding pioneer species usually grows faster than shade-tolerant species if conditions are favorable. These physiological differences have an effect on tree diameters. For this reason, the correlations between *dbh*'s and soil chemical variables may be meaningless. Thus, the spatial characteristics of tree diameters may be interpreted by the variations of topography and soil chemical variables to some degree, but the underlying causes are not always obvious. Soil chemistry might give some species an advantage over others, but small-scale neighboring competition and historical gap formation are likely to have a significant influence.

## Conclusion

The major objective of this study was to improve our knowledge about the spatial correlations between tree diameters. Based on a large dataset of mapped trees, our research has shown that the diameters of most species are negatively correlated in space. The percentage of species showing significant *dbh* suppression peaked at short distances especially. In the beginning of the 20th century, the original virgin forest has been subjected to large-scale industrial logging, and then replaced by a secondary forests and plantations [43]. During the past 60 years, the protection of these unique ecosystems has been ensured and is still a matter of great concern to ecologist and local foresters.

However, it may be possible that selective harvest in continuous cover forest management systems [44] will be introduced in the future. In that case, our research may provide some guidance to local management. This study has shown that neighboring competition effects which may reduce the growth of valuable target trees, can be avoided if competing individuals are removed within a radius of less 10 m around the target individuals. However, this result could be merely a local effect, involving the particular species community in the observational study area and further research in other ecosystems is required to obtain a broader understanding of competition effects in different communities.

## Supporting Information

Figure S1 **Soil sampling map in the old-growth forest plot.** Grids are 40 m×40 m and 40 m×20 m. The intersections of 40 m×40 m grid lines are regarded as base points (Black points). Based on theses base points, we randomly selected one of eight directions to sample at 2 m and 8 m, or 2 m and 15 m or 8 m and 15 m from base points. Red points are 2-m extra points, blue points are 8-m extra points and green points are 15-m extra points. Total 540 sample points were determined in the research plot.
(DOCX)

Figure S2 **Spatial distributions of size differentiation and topographic variables.** The shading from light to dark shows an increase from low (0.6537) to high (2.0699) size differentiation, from low (577.8) to high (780.7) elevation (m), from low (2.4) to high (45.7) slope (degrees), from low (72.8) to high (299.9) aspect (degrees) and from low (−6.8) to high (8.6) convexity.
(DOCX)

Figure S3 **The image and contour plots for soil chemical properties.**
(DOCX)

Figure S4 **Semi-variogram maps of soil chemical properties.**
(DOCX)

Table S1 **The list of growth forms and the number of individuals for examined species in the 30-ha OGF plot.**
(DOCX)

Table S2 **Data transform based on parameter estimation for the Box-Cox transformation.**
(DOCX)

Table S3 **Semivariogram models of soil chemical properties in old-growth forest plot.**
(DOCX)

Table S4 **Person correlation coefficients between tree diameter and soil chemical properties.**
(DOCX)

Text S1 **The main codes used in our analyses.**
(DOCX)
