# Supplementary material for: Spatial Characteristics of Tree Diameter Distributions in a Temperate Old-Growth Forest
Source: PLoS One. 2013 Mar 19;8(3):e58983. doi: 10.1371/journal.pone.0058983 (PMC3602579; doi:10.1371/journal.pone.0058983)

**Figure S1:** Soil sampling map in the old-growth forest plot. Grids are 40m×40m and 40m×20m. The intersections of 40m×40m grid lines are regarded as base points (Black points). Based on theses base points, we randomly selected one of eight directions to sample at 2m and 8m, or 2m and 15m or 8m and 15m from base points. Red points are 2-m extra points, blue points are 8-m extra points and green points are 15-m extra points. Total 540 sample points were determined in the research plot.


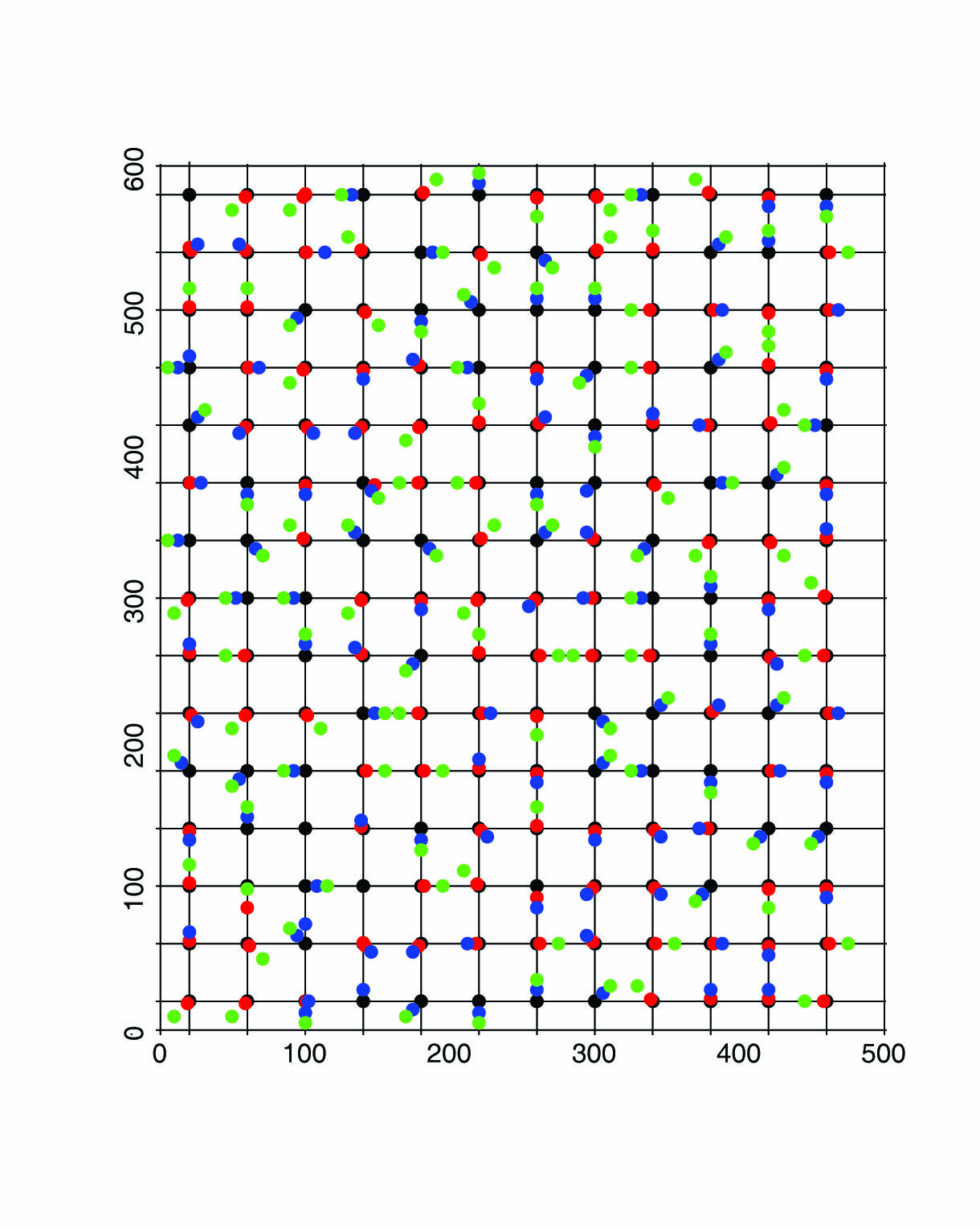

Supplement: Figure S1 — Soil sampling map in the old-growth forest plot. Grids are 40 m×40 m and 40 m×20 m. The intersections of 40 m×40 m grid lines are regarded as base points (Black points). Based on theses base points, we randomly selected one of eight directions to sample at 2 m and 8 m, or 2 m and 15 m or 8 m and 15 m from base points. Red points are 2-m extra points, blue points are 8-m extra points and green points are 15-m extra points. Total 540 sample points were determined in the research plot. (DOCX) [file pone.0058983.s001.docx]
