# Supplementary material for: Spatial Characteristics of Tree Diameter Distributions in a Temperate Old-Growth Forest
Source: PLoS One. 2013 Mar 19;8(3):e58983. doi: 10.1371/journal.pone.0058983 (PMC3602579; doi:10.1371/journal.pone.0058983)
Supplement: Figure S2 — Spatial distributions of size differentiation and topographic variables. The shading from light to dark shows an increase from low (0.6537) to high (2.0699) size differentiation, from low (577.8) to high (780.7) elevation (m), from low (2.4) to high (45.7) slope (degrees), from low (72.8) to high (299.9) aspect (degrees) and from low (−6.8) to high (8.6) convexity. (DOCX) [file pone.0058983.s002.docx]

**Figure S2:** Spatial distributions of size differentiation and topographic variables. The shading from light to dark shows an increase from low (0.6537) to high (2.0699) size differentiation, from low (577.8) to high (780.7) elevation (m), from low (2.4) to high (45.7) slope (degrees), from low (72.8) to high (299.9) aspect (degrees) and from low (-6.8) to high (8.6) convexity.

| A Size differentiation | B Elevation | C Slope |
| --- | --- | --- |
|  |  |  |
| D Aspect | E Convexity |  |
|  |  |  |
