# Supplementary material for: Spatial Characteristics of Tree Diameter Distributions in a Temperate Old-Growth Forest
Source: PLoS One. 2013 Mar 19;8(3):e58983. doi: 10.1371/journal.pone.0058983 (PMC3602579; doi:10.1371/journal.pone.0058983)
Supplement: Figure S4 — Semi-variogram maps of soil chemical properties. (DOCX) [file pone.0058983.s004.docx]

**Figure S4:** Semi-variogram maps of soil chemical properties.

| A Total N in upper layer | B Total N in middle layer | C Total N in lower layer |
| --- | --- | --- |
|  |  |  |
| D Total K in upper layer | E Total K in middle layer | F Total K in lower layer |
|  |  |  |
| G pH in upper layer | H pH in middle layer | I pH in lower layer |
|  |  |  |
| J Organic matter in upper layer | K Organic matter in middle layer | L Organic matter in lower layer |
| **** | **** | **** |
