# Supplementary material for: Spatial Characteristics of Tree Diameter Distributions in a Temperate Old-Growth Forest
Source: PLoS One. 2013 Mar 19;8(3):e58983. doi: 10.1371/journal.pone.0058983 (PMC3602579; doi:10.1371/journal.pone.0058983)
Supplement: Table S1 — The list of growth forms and the number of individuals for examined species in the 30-ha OGF plot. (DOCX) [file pone.0058983.s005.docx]

**Supporting Information Table 1:**

**The list of growth forms and the number of individuals for examined species in the 30-ha OGF plot**

| **Species name** | **Family** | **Growth forms** | **Number of individuals** |
| --- | --- | --- | --- |
| *Betula platyphylla* | Betulaceae | tree | 71 |
| *Acer mandshuricum* | Aceraceae | tree | 5447 |
| *Syringa reticulata* var. *amurensis* | Oleaceae | small tree | 3025 |
| *Euonymus macropterus* | Celastraceae | shrub | 898 |
| *Padus racemosa* | Rosaceae | small tree | 722 |
| *Abies nephrolepis* | Pinaceae | tree | 233 |
| *Ulmus davidiana* var. *japonica* | Ulmaceae | tree | 94 |
| *Acanthopanax senticosus* | Araliaceae | shrub | 30 |
| *Acer barbinerve* | Aceraceae | small tree | 6937 |
| *Ulmus macrocarpa* | Ulmaceae | tree | 213 |
| *Philadelphus schrenkii* | Saxifragaceae | shrub | 20 |
| *Betula costata* | Betulaceae | tree | 829 |
| *Betula dahurica* | Betulaceae | tree | 76 |
| *Cerasus maximowiczii* | Rosaceae | small tree | 293 |
| *Pinus koraiensis* | Pinaceae | tree | 1339 |
| *Juglans mandshurica* | Juglandaceae | tree | 834 |
| *Acer ukurunduense* | Aceraceae | small tree | 2058 |
| Sorbus pohuashanensis | Rosaceae | small tree | 61 |
| *Fraxinus rhynchophylla* | Oleaceae | tree | 26 |
| *Phellodendron amurense* | Rutaceae | tree | 824 |
| *Lonicera praeflorens* | Caprifoliaceae | shrub | 23 |
| *Lonicera maackii* | Caprifoliaceae | shrub | 38 |
| *Tilia mandshurica* | Tiliaceae | tree | 110 |
| *Ulmus laciniata* | Ulmaceae | tree | 2813 |
| *Euonymus pauciflorus* | Celastraceae | shrub | 418 |
| *Aralia elata* | Araliaceae | shrub | 28 |
| *Corylus mandshurica* | Betulaceae | shrub | 5387 |
| *Quercus mongolica* | Fagaceae | tree | 151 |
| *Carpinus cordata* | Betulaceae | tree | 6682 |
| *Acer tegmentosum* | Aceraceae | small tree | 1818 |
| *Acer mono* | Aceraceae | tree | 4112 |
| *Abies holophylla* | Pinaceae | tree | 464 |
| *Rhamnus davurica* | Rhamnaceae | small tree | 79 |
| *Fraxinus mandshurica* | Oleaceae | tree | 408 |
| *Sorbus alnifolia* | Rosaceae | tree | 1065 |
| *Populus koreana* | Salicaceae | tree | 41 |
| *Tilia amurensis* | Tiliaceae | tree | 1962 |
