# Supplementary material for: Spatial Characteristics of Tree Diameter Distributions in a Temperate Old-Growth Forest
Source: PLoS One. 2013 Mar 19;8(3):e58983. doi: 10.1371/journal.pone.0058983 (PMC3602579; doi:10.1371/journal.pone.0058983)
Supplement: Table S2 — Data transform based on parameter estimation for the Box-Cox transformation. (DOCX) [file pone.0058983.s006.docx]

**Supporting Information Table 2:**

**Data transform based on parameter estimation for the Box-Cox transformation**

| **Soil properties** | **Box-Cox transformation** |
| --- | --- |
| Total N-Upper | x’= (x^0.26061959^-1)/ 0.26061959 |
| Total N-Middle | x’= (x^-0.09150445^-1)/ (-0.09150445) |
| Total N-Lower | x’= (x^0.006019725^-1)/ 0.006019725 |
| Total K-Upper | x’= (x^0.5065252^-1)/ 0.5065252 |
| Total K-Middle | x’= (x^0.2477724^-1)/ 0.2477724 |
| Total K-Lower | x’= (x ^-0.254034733^-1)/ (-0.254034733) |
| pH-Upper | x’= (x^1.952205^-1)/ 1.952205 |
| pH-Middle | x’= (x^0.5365134^-1)/ 0.5365134 |
| pH-Lower | x’= (x^-0.306298279^-1)/ (-0.306298279) |
| OM-Upper | x’= (x^0.423287^ -1)/ 0.423287 |
| OM-Middle | x’= (x^-0.00485634^ -1)/ (-0.00485634) |
| OM-Lower | x’= (x^0.2467412^ -1)/ 0.2467412 |
