# Supplementary material for: Spatial Characteristics of Tree Diameter Distributions in a Temperate Old-Growth Forest
Source: PLoS One. 2013 Mar 19;8(3):e58983. doi: 10.1371/journal.pone.0058983 (PMC3602579; doi:10.1371/journal.pone.0058983)
Supplement: Table S3 — Semivariogram models of soil chemical properties in old-growth forest plot. (DOCX) [file pone.0058983.s007.docx]

**Supporting Information Table 3:**

**Semivariogram models of soil chemical properties in old-growth forest plot**

| **Soil properties** | **Model** | **Nugget (C_0_)** | **Sill (C)** | **Range (phi)** | **C/(C_0_+C)** |
| --- | --- | --- | --- | --- | --- |
| Total N-Upper | exponential | 0.08 | 0.02 | 56.68 | 0.2 |
| Total N-Middle | gaussian | 0.11 | 0.04 | 38.98 | 0.27 |
| Total N-Lower | exponential | 0.09 | 0.07 | 46.77 | 0.44 |
| Total K-Upper | exponential | 4.28 | 3.5 | 171.51 | 0.41 |
| Total K-Middle | exponential | 0.42 | 0.24 | 23.39 | 0.36 |
| Total K-Lower | gneiting | 0.40 | 0.28 | 241.67 | 0.41 |
| pH-Upper | exponential | 1.55 | 3.37 | 15.59 | 0.68 |
| pH-Middle | exponential | 0.038 | 0.01 | 23.39 | 0.21 |
| pH-Lower | exponential | 0.002 | 0.001 | 32 | 0.33 |
| OM-Upper | exponential | 1516.5 | 1299.86 | 140.32 | 0.46 |
| OM-Middle | exponential | 0.175 | 0.13 | 296.24 | 0.43 |
| OM-Lower | exponential | 1.27 | 1.02 | 70.16 | 0.45 |
