# Supplementary material for: Spatial Characteristics of Tree Diameter Distributions in a Temperate Old-Growth Forest
Source: PLoS One. 2013 Mar 19;8(3):e58983. doi: 10.1371/journal.pone.0058983 (PMC3602579; doi:10.1371/journal.pone.0058983)
Supplement: Table S4 — Person correlation coefficients between tree diameter and soil chemical properties. (DOCX) [file pone.0058983.s008.docx]

**Supporting Information Table 4:**

**Person correlation coefficients between tree diameter and soil chemical properties**

| **Species name** | **Total N** | | | **Total K** | | | **pH** | | | **Organic matter** | | |
| --- | --- | --- | --- | --- | --- | --- | --- | --- | --- | --- | --- | --- |
|  | Upper | Middle | Lower | Upper | Middle | Lower | Upper | Middle | Lower | Upper | Middle | Lower |
| *Betula platyphylla* | -0.28^*^ | -0.20 | -0.24^*^ | 0.07 | 0.12 | 0.00 | -0.04 | -0.07 | 0.23 | 0.04 | -0.03 | -0.15 |
| *Acer mandshuricum* | -0.1^***^ | -0.04^**^ | -0.05^***^ | 0.07^***^ | 0.09^***^ | 0.00 | -0.04^***^ | -0.07^***^ | -0.06^***^ | -0.05^*^ | -0.04^**^ | 0 |
| *Syringa reticulata* var. *amurensis* | -0.03 | -0.02 | 0.11^***^ | 0.10^***^ | 0.03 | -0.19^***^ | -0.05^**^ | -0.02 | 0.01 | -0.17^***^ | -0.15^***^ | -0.13^***^ |
| *Euonymus macropterus* | -0.05 | 0.03 | 0.08^*^ | 0.11^**^ | -0.01 | -0.10^**^ | -0.09^**^ | -0.07^*^ | -0.01 | -0.12^***^ | -0.06 | -0.09^**^ |
| *Padus racemosa* | 0.1^**^ | 0.13^***^ | 0.13^***^ | 0.08^*^ | -0.04 | -0.02 | -0.12^**^ | -0.09^*^ | -0.18^***^ | -0.19^***^ | -0.16^***^ | -0.08^*^ |
| *Abies nephrolepis* | 0.01 | 0.00 | 0.05 | 0.20^**^ | 0.22^***^ | -0.13^*^ | 0.15^**^ | -0.07 | 0.02 | -0.17^**^ | -0.17^**^ | -0.13^*^ |
| *Ulmus davidiana* var. *japonica* | -0.13 | -0.12 | 0.09 | 0.18 | 0.01 | -0.20 | -0.15 | -0.2^*^ | 0.02 | -0.04 | -0.08 | -0.08 |
| *Acanthopanax senticosus* | 0.48^**^ | 0.32 | -0.10 | -0.34 | -0.49^**^ | 0.35 | 0.17 | 0.08 | -0.39^*^ | 0.35 | 0.12 | 0.20 |
| *Acer barbinerve* | -0.03^**^ | 0.00 | 0.03^*^ | 0.08^***^ | 0.05^***^ | -0.05^***^ | -0.01 | 0.01 | 0.02 | -0.07^***^ | -0.06^***^ | -0.05^***^ |
| *Ulmus macrocarpa* | -0.21^**^ | 0.03 | 0.15^*^ | 0.29^***^ | 0.24^***^ | -0.22^**^ | -0.17^*^ | -0.06 | -0.24^***^ | -0.22^**^ | -0.24^***^ | 0.26^***^ |
| *Philadelphus schrenkii* | -0.51^*^ | -0.40 | 0.11 | 0.34 | 0.36 | -0.26 | 0.06 | 0.11 | 0.30 | 0.03 | -0.14 | 0.06 |
| *Betula costata* | 0.05 | 0.06 | 0.13^***^ | -0.14^***^ | -0.20^***^ | -0.03 | -0.05 | 0.01 | 0.02 | 0.09^**^ | 0.15^***^ | 0.02 |
| *Betula dahurica* | 0.13 | -0.15 | 0.01 | -0.18 | -0.19 | 0.18 | -0.30^**^ | -0.20 | -0.15 | 0.07 | 0.03 | 0.00 |
| *Cerasus maximowiczii* | -0.09 | -0.07 | -0.07 | 0.25^***^ | 0.17^**^ | -0.04 | -0.04 | 0.11 | 0.14^*^ | -0.15^**^ | -0.17^**^ | -0.03 |
| *Pinus koraiensis* | 0.08^**^ | 0.16^***^ | 0.09^**^ | 0.15^***^ | 0.03 | -0.12^***^ | -0.17^***^ | -0.08^**^ | -0.04 | -0.15^***^ | -0.13^***^ | -0.11^***^ |
| *Juglans mandshurica* | 0.14^***^ | 0.02 | -0.03 | -0.16^***^ | 0.12^***^ | 0.01 | 0.05 | 0.01 | 0.04 | 0.05 | 0.03 | 0.05 |
| *Acer ukurunduense* | -0.09^***^ | -0.04 | -0.05^*^ | 0.09^***^ | 0.09^***^ | -0.01 | -0.02 | -0.02 | -0.02 | -0.03 | -0.01 | 0.00 |
| *Sorbus pohuashanensis* | -0.04 | 0.15 | 0.03 | 0.06 | 0.28^*^ | 0.03 | 0.17 | 0.09 | 0.28^*^ | 0.05 | 0.07 | 0.09 |
| *Fraxinus rhynchophylla* | 0.66^***^ | 0.06 | -0.23 | -0.32 | 0.40 | 0.49 | 0.05 | 0.15 | 0.21 | 0.34 | 0.21 | 0.18 |
| *Phellodendron amurense* | 0.07^*^ | 0.13^***^ | 0.16^***^ | -0.05 | -0.14^***^ | -0.13^***^ | -0.06 | 0.00 | 0.05 | -0.05 | -0.03 | -0.06 |
| *Lonicera praeflorens* | -0.38 | -0.05 | 0.17 | -0.12 | -0.33 | -0.33 | 0.30 | 0.05 | 0.03 | 0.08 | -0.12 | 0.23 |
| *Lonicera maackii* | -0.23 | -0.18 | 0.00 | 0.00 | -0.17 | 0.15 | -0.10 | 0.22 | 0.32^*^ | 0.30 | 0.37^*^ | 0.19 |
| *Tilia mandshurica* | 0.00 | 0.14 | 0.11 | -0.01 | -0.15 | -0.12 | -0.14 | 0.02 | 0.03 | -0.11 | -0.11 | -0.19^*^ |
| *Ulmus laciniata* | 0.00 | 0.06^**^ | 0.08^***^ | 0.04^*^ | -0.04^*^ | -0.06^***^ | -0.07 | -0.01 | -0.09^***^ | -0.03 | 0.01 | -0.07 |
| *Euonymus pauciflorus* | -0.13^**^ | 0.06 | 0.08 | 0.11^*^ | 0.04 | -0.21^***^ | -0.10^*^ | -0.09 | 0.07 | -0.14 | -0.14^**^ | -0.11^*^ |
| *Aralia elata* | 0.00 | 0.03 | 0.04 | 0.21 | 0.12 | -0.14 | -0.13 | -0.39^*^ | -0.23 | -0.23 | -0.14 | -0.19 |
| *Corylus mandshurica* | 0.03^*^ | 0.04^**^ | 0.04^**^ | 0.03^*^ | -0.01 | -0.03^*^ | 0.03^*^ | 0.05^***^ | 0.02 | -0.04^**^ | -0.04 | -0.05^***^ |
| *Quercus mongolica* | -0.05 | -0.09 | 0.00 | -0.03 | 0.05 | 0.13 | -0.18^*^ | -0.25^**^ | -0.21^**^ | 0.16^*^ | 0.22^**^ | 0.16^*^ |
| *Carpinus cordata* | 0.10^***^ | 0.08^***^ | 0.00 | 0.05^***^ | 0.01 | 0.00 | 0.00 | 0.01 | -0.04^**^ | -0.1^***^ | -0.11^***^ | -0.13^***^ |
| *Acer tegmentosum* | 0.02 | 0.04 | -0.01 | 0.09^***^ | 0.01 | -0.06^**^ | -0.04 | -0.02 | -0.08^***^ | -0.05 | -0.07^**^ | -0.04 |
| *Acer mono* | 0.03 | 0.05^***^ | 0.00 | -0.06^***^ | -0.08^***^ | 0.00 | -0.07^***^ | -0.07^***^ | -0.05^***^ | 0.03 | 0.05^***^ | 0.02 |
| *Abies holophylla* | 0.00 | 0.00 | 0.06 | 0.05 | 0.05 | -0.03 | -0.02 | -0.01 | 0.10^*^ | -0.07 | -0.08 | -0.07 |
| *Rhamnus davurica* | 0.15 | 0.15 | 0.01 | 0.24^*^ | 0.00 | 0.08 | -0.11 | -0.08 | -0.03 | -0.12 | -0.28^*^ | -0.18 |
| *Fraxinus mandshurica* | -0.04 | -0.10^*^ | -0.06 | -0.06 | -0.14^**^ | 0.09 | 0.05 | 0.02 | -0.01 | 0.12^*^ | 0.10 | 0.04 |
| *Sorbus alnifolia* | 0.02 | 0.09^**^ | -0.02 | 0.12^***^ | 0.11^***^ | 0.01 | -0.03 | 0.00 | -0.07^*^ | -0.08^**^ | -0.07^*^ | -0.09^**^ |
| *Populus koreana* | -0.11 | 0.01 | 0.40^*^ | 0.51^***^ | 0.23 | -0.27 | 0.03 | 0.00 | 0.38^*^ | 0.39^*^ | -0.37^*^ | -0.06 |
| *Tilia amurensis* | 0.03 | 0.04 | 0.04 | -0.06^**^ | -0.05^*^ | 0.07^**^ | 0.07 | 0.08^***^ | 0.08^***^ | 0.07^**^ | 0.08^***^ | 0.03 |
