# Supplementary material for: Spatial Characteristics of Tree Diameter Distributions in a Temperate Old-Growth Forest
Source: PLoS One. 2013 Mar 19;8(3):e58983. doi: 10.1371/journal.pone.0058983 (PMC3602579; doi:10.1371/journal.pone.0058983)
Supplement: Text S1 — The main codes used in our analyses. (DOCX) [file pone.0058983.s009.docx]

# This program is to compute the confidence intervals based on the models of HomP,

# RLM and HetP.

# simulate homogeneous Poisson process

HomP.fn= function(data, sp, xlength,ylength) {

###################

# sp: character. the calculated species;

# xlength: numeric. the x-axis length of plot;

# ylength: numeric. the y-axis length of plot.

####################

library(spatstat)

x=data[data$species==sp,]$x

y= data [data $species==sp,]$y

z= data [data $species==sp,]$dbh

data1=data.frame(x=x,y=y,z=z)

w <- owin(c(0, xlength),c(0, ylength))

data.ppp=as.ppp(data1,w)

E=envelope(data.ppp, markcorr)

return(E)

}

# simulate heterogeneous Poisson model

HetP.fn= function(data, sp, xlength,ylength) {

###################

# sp: character. the calculated species;

# xlength: numeric. the x-axis length of plot;

# ylength: numeric. the y-axis length of plot.

####################

library(spatstat)

x=data[data $species==sp,]$x

y= data [data $species==sp,]$y

z= data [data $species==sp,]$dbh

data1=data.frame(x=x,y=y,z=z)

w <- owin(c(0, xlength),c(0, ylength))

data.ppp=as.ppp(data1,w)

MCF=markcorr(data.ppp, correction="isotropic", method="density", kernel="epanechnikov")

trend <- density.ppp(data.ppp, bw.diggle)

envel=numeric()

for (i in 1:99) {

coor=rpoispp(trend)

xx= coor[[3]]

yy= coor[[4]]

zz=sample(z,coor[[2]], replace =T)

datasim=data.frame(x=xx,y=yy,z=zz)

w <- owin(c(0, xlength),c(0, ylength))

datasim.ppp=as.ppp(datasim,w)

MCFsim=markcorr(datasim.ppp, correction="isotropic", method="density", kernel="epanechnikov")

envel=cbind(envel, MCFsim$iso)

}

maxvalue= numeric()

minvalue= numeric()

for(j in 1:dim(envel)[1]){

maxvalue[j]=max(envel[j,])

minvalue[j]=min(envel[j,])

}

HetPoisMCF= data.frame(MCF$r ,MCF$iso, maxvalue, minvalue)

return(HetPoisMCF)

}

# simulate random labeling model

RLM.fn= function(data, sp, xlength,ylength) {

###################

# sp: character. the calculated species;

# xlength: numeric. the x-axis length of plot;

# ylength: numeric. the y-axis length of plot.

####################

library(spatstat)

x= data [data $species==sp,]$x

y= data [data $species==sp,]$y

z= data [data $species==sp,]$dbh

data1=data.frame(x=x,y=y,z=z)

w <- owin(c(0, xlength),c(0, ylength))

data.ppp=as.ppp(data1,w)

MCF=markcorr (data.ppp, correction="isotropic", method="density", kernel="epanechnikov")

envel=numeric()

for (i in 1:99) {

zz=sample(z)

datasim=data.frame(x=x,y=y,z=zz)

w <- owin(c(0, xlength),c(0, ylength))

datasim.ppp=as.ppp(datasim,w)

MCFsim=markcorr(datasim.ppp, correction="isotropic", method="density", kernel="epanechnikov")

envel=cbind(envel, MCFsim$iso)

rm(zz);rm(datasim);rm(w);rm(datasim.ppp);rm(MCFsim)

}

maxvalue= numeric()

minvalue= numeric()

for(j in 1:dim(envel)[1]){

maxvalue[j]=max(envel[j,])

minvalue[j]=min(envel[j,])

}

RLMCF= data.frame(MCF$r ,MCF$iso, maxvalue, minvalue)

return(RLMCF)

}
